# Nanometer-Resolved Operando Photo-Response of Faceted BiVO_4_ Semiconductor Nanoparticles

**DOI:** 10.1021/jacs.3c12666

**Published:** 2024-01-12

**Authors:** Shaoqiang Su, Igor Siretanu, Dirk van den Ende, Bastian Mei, Guido Mul, Frieder Mugele

**Affiliations:** † Physics of Complex Fluids Group and MESA+ Institute, Faculty of Science and Technology, University of Twente, P.O. Box 217, Enschede 7500 AE, The Netherlands; ‡ Photocatalytic Synthesis Group and MESA+ Institute, Faculty of Science and Technology, University of Twente, P.O. Box 217, Enschede 7500 AE, The Netherlands

## Abstract

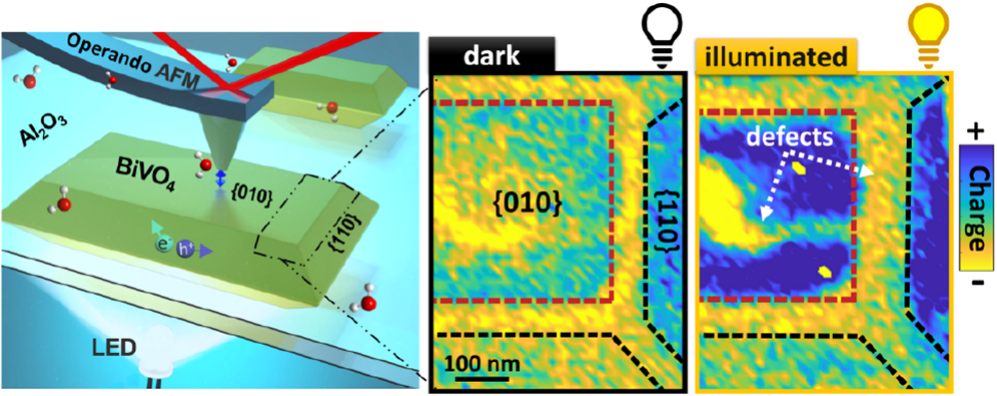

Photo(electro)catalysis with semiconducting nanoparticles (NPs) is an attractive approach to convert abundant but intermittent renewable electricity into stable chemical fuels. However, our understanding of the microscopic processes governing the performance of the materials has been hampered by the lack of operando characterization techniques with sufficient lateral resolution. Here, we demonstrate that the local surface potentials of NPs of bismuth vanadate (BiVO_4_) and their response to illumination differ between adjacent facets and depend strongly on the pH of the ambient electrolyte. The isoelectric points of the dominant {010} basal plane and the adjacent {110} side facets differ by 1.5 pH units. Upon illumination, both facets accumulate positive charges and display a maximum surface photoresponse of +55 mV, much stronger than reported in the literature for the surface photo voltage of BiVO_4_ NPs in air. High resolution images reveal the presence of numerous surface defects ranging from vacancies of a few atoms, to single unit cell steps, to microfacets of variable orientation and degree of disorder. These defects typically carry a highly localized negative surface charge density and display an opposite photoresponse compared to the adjacent facets. Strategies to model and optimize the performance of photocatalyst NPs, therefore, require an understanding of the distribution of surface defects, including the interaction with ambient electrolyte.

## Introduction

Photocatalysis is a promising approach to convert intermittent solar energy to stable chemical fuels.^1,2^ Like its natural counterpart photosynthesis, it combines the absorption of light and the synthesis of a stable chemical product in individual nanoscale units, typically functionalized faceted semiconductor nanoparticles (NPs).^3−6^ Next to generating H_2_ by water splitting, photocatalysis also allows to synthesize other, more valuable hydrocarbons from water and CO_2_ and, in other contexts, to decompose undesirable solutes such as organic contaminants in drinking water. In all cases, the efficient separation of photogenerated electron–hole pairs and their transfer to the reaction sites at the surface of the NP are essential for the overall performance of the process.^3−6^ In this respect, the introduction of anisotropic faceted NPs has led to substantial improvements, because the difference in surface potentials between different crystallographic facets gives rise to electric fields within the NPs that separate electrons and holes similar to p–n junctions in photovoltaic cells.^3−12^ However, given the large surface-to-volume ratio of photocatalyst NPs and the strong interaction with the ambient electrolyte, the local potentials are controlled not only by the properties of the semiconductor but also by its surroundings, i.e. by the chemistry of the solid-electrolyte interface.^13−16^ For instance, common oxidic materials display hydroxyl groups at the surface that can become (de)protonated and complex with ions from solution depending on the fluid composition. Since such surface speciation reactions are generally facet-dependent, the difference in surface potential between adjacent facets and thus the electric fields driving the separation of photogenerated charge carriers also becomes a function of the electrolyte composition.^17−20^ In addition to different facets, surface defects also display a different local surface chemistry and hence presumably different local surface potentials that can affect the separation of charge carriers.^4,7,21−23^

A quantitative analysis and understanding of photocatalytic NPs, therefore, require in the first place experimental tools that allow for a nanometer-resolved characterization under operating conditions, i.e., in ambient electrolyte at variable illumination.^24−26^ Classical surface science techniques (e.g., XPS, vibrational spectroscopy) do not offer the required spatial resolution.^16,24,25^ Scanning probe-based techniques such as surface photovoltage microscopy and Kelvin probe force microscopy have provided valuable insights into the facet-dependent surface photo voltage (SPV).^12,27−29^ Yet, such experiments are usually carried out in air and may not be easily transferrable to actual operating conditions in ambient liquid.^17,30−33^ In the absence of microscopic in situ characterization, models describing the response of photocatalysts under illumination typically neglect the ambient electrolyte and instead treat the unknown surface potentials as fit parameters.^9,34^ While providing a reasonable description of the macroscopic photo/electrocatalytic performance, important microscopic aspects remain unresolved, including the pH dependence of the photocatalytic performance and the effect of lateral heterogeneities within the facets. In a recent work, it was concluded that photoexcited charge carriers at facet surfaces behave fundamentally differently from classical semiconductor theory and instead act as independent two-dimensional electronic systems decoupled from the bulk of the NP with very poor electrostatic screening capabilities.^35^ This surprising result would obviously have important consequences for the design of photocatalysts including their optimum shape and the distribution of cocatalysts that distort the electronic band structure in order to improve charge carrier transfer.^4,5,32,36^

In order to quantify the local distribution of the surface potentials, we recently applied our dual scale atomic force microscopy (AFM)-based method^37−41^ that combines AFM probes of different size to characterize local surface charge, hydration, and chemistry to photocatalytically active NPs of SrTiO_3_.^17^ We demonstrated a strong facet- and pH-dependent variation of the local surface charge density and corresponding local surface potential.^17^ In the present work, we extend these previous *in situ* measurements to *operando* conditions and measure the photoresponse of NPs of visible light-responsive BiVO_4_, a material that is widely used as an oxygen evolution catalyst.^10,12,42,43^ Upon illumination, the electrically isolated particles are found to charge up positively with respect to the ambient electrolyte with a strongly facet- and pH-dependent photoresponse of the local surface potential. Our measurements reveal an important contribution of surface defects, which are rather widespread on BiVO_4_. These defects display sharp contours and a photoresponse that exceeds the one of crystalline facets up to three times. Our results highlight the relevance of both surface defects and the composition of the ambient electrolyte for the photoresponse of photocatalytic NPs and provide suggestions how these insights can be used to optimize the performance of photocatalytic systems.

## Results and Discussion

### Facet-Dependent Surface Charge Density Measurement

**Figure 1:**
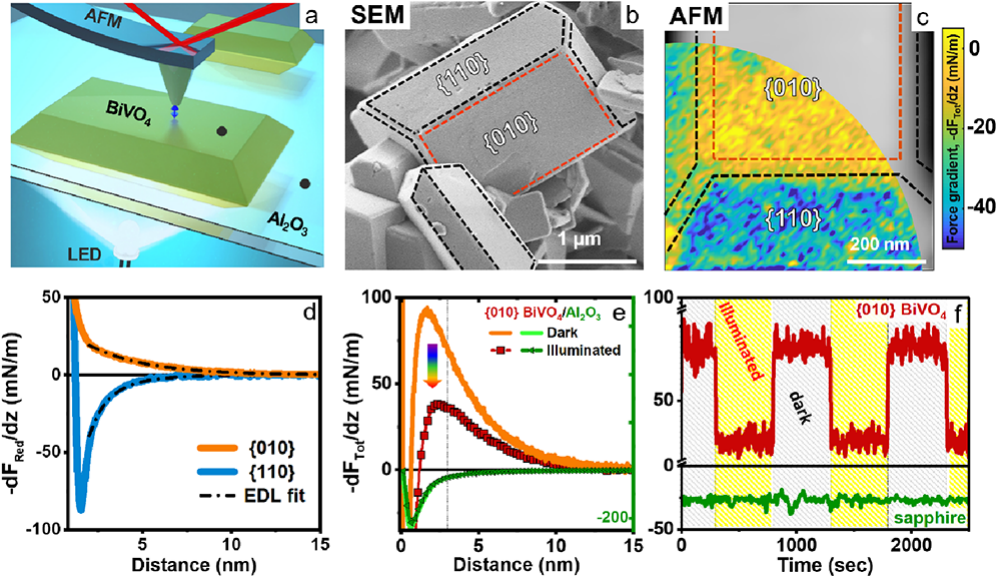
Atomic force microscopy (AFM) measurement on BiVO_4_ particles and the photoresponse in ambient electrolyte. a) Illustration of dynamic AFM measurements in liquid with bottom illumination. b) SEM image of BiVO_4_ nanoparticles. Red and black shapes indicate the {010} and {110} facets. c) Height channel of the AFM topography measurement on a BiVO_4_ NP, left half, is superimposed by the corresponding 2D force gradient (−dF_Tot_/dz) map measured in 10 mM NaCl with a pH of 5.8. The 2D map was extracted from a 3D force versus distance volume plot (60 × 60 interaction curves) when the tip is 2.5 nm away from BiVO_4_. AFM tip radius = 27 ± 1 nm. Color code: yellow and blue colors indicate repulsive and attractive interactions, respectively. d) Average reduced interaction stiffness (*k*_red_) or force gradient (−dF_red_/dz) versus distance curves across a flat region at the center of {010} and {110} facets of BiVO_4_ particles. Solid lines are experimental data after subtraction of van der Waals interaction (*k*_Tot_–*k*_vdW_) and dashed–dotted black lines are the theoretically fitted force curves using EDL theory, taking into account charge regulation. The total interaction stiffness (*k*_Tot_) or total force gradient (−dF_Tot_/dz) are shown in Figure S3. e) Average force gradient (*k*_Tot_) versus distance curves obtained on one single point of {010} facet of BiVO_4_ (right Y axis) and sapphire (Al_2_O_3_) substrate (green left Y axis) in the dark and under illumination. The average is based on forces measured during an 8 min period. f) Force gradient values at 3 nm away from {010} of BiVO_4_ and Al_2_O_3_ (dotted line in panel e), collected during alternating illumination and dark spans. AFM tip radius = 22.6 ± 1 nm. Corresponding NP topography images and extra information are shown in Figures S2 and S4 Supporting Information.

Faceted bismuth vanadate (BiVO_4_) NPs are synthesized using the hydrothermal method^10^ (see also the Experimental Section). The BiVO_4_ particles display the expected monoclinic structure and decahedral shape with two large square or slightly rectangular {010} facets each surrounded by four trapezoid facets, typically assigned as {110} facets (Figures 1b and S1). Our BiVO_4_ particles display a width of 0.5–2 μm and thicknesses ranging from several tens to a few hundred nanometers, as observed by both scanning electron microscopy (SEM) and AFM. The total surface area of the basal planes is similar to the one of all the trapezoidal facets combined. For AFM characterization, the particles are immobilized on clean sapphire (Al_2_O_3_) substrates by spontaneous adsorption from an aqueous suspension (see the Experimental Section).

To characterize the local surface charge of the different facets, AFM spectroscopy measurements are performed on NPs immersed in aqueous solutions of NaCl (concentration: 10 mM) at variable pH.^17,38^ AFM images and maps of the force gradient display the topography and provide access to the local surface charges of the different facets (Figure 1c). Data recorded at pH 5.8 display a repulsive force gradient on the {010} facets (yellow in Figure 1c) and an attractive force gradient on the {110} side facets (blue-cyan). Since the surface of the AFM tip is made of oxidized silicon, which is negatively charged for all pH values in the present study, this implies that the {010} facet is negatively charged, while the {110} facets carry a positive charge. Averages over homogeneous areas on each facet allow to extract representative curves of the force gradient vs distance (Figure 1d), from which we extract the charge densities per facet using established fitting procedures based on Poisson–Boltzmann theory (dashed–dotted black lines in Figure 1d; see also the Experimental Section). The force sensed by the AFM and thus the extracted values of the surface charge density σ arise from the ions in the diffuse part of the electric double layer^41^ (see the Experimental Section for details). For the conditions shown in Figure 1b,c, the local surface potential amounts to −48 mV for the {010} facet and to +18 mV for the {110} facet. Hence, there is a total potential difference of 66 mV between the two adjacent facets in the dark for the present electrolyte composition. This value is substantially higher than the few mV typically reported for bare BiVO_4_ particles in air or vacuum based on Kelvin probe force microscopy (KPFM) and spatially resolved surface (photo)voltage spectroscopy (SRSPS).^27−29^ Unless illumination induces substantial changes in the compact part of the electric double layer, such as ion adsorption and the orientation of the water dipole, this stronger photoresponse suggests that the interaction with the ambient fluid can dramatically enhance the potential difference between the adjacent facets and thereby the driving force for separating electrons and holes in the semiconductor.^6^

### Facet-Dependent Photoresponse in Ambient Electrolyte

To explore the response of the surface potential to light, we illuminate the adsorbed BiVO_4_ NPs from below through the transparent substrate with a broadband light emitting diode (LED) with a wavelength range of 420–680 nm, Figure 1a (see also the Experimental Section). The bandgap of BiVO_4_ of 2.3–2.5 eV corresponds to λ_g_ = 500–550 nm.^4,42^ In all experiments, illumination reduces the electrostatic repulsion on the facets of the NPs, as illustrated for {010} in Figure 1e. This indicates a reduction of the negative surface charge density, consistent with the reported upward band bending at both the {010} and the {110} surface. Repeated switching of the light source demonstrates the reversibility of the photo response (Figure 1f top panel). Control measurements on the adjacent sapphire (Al_2_O_3_) substrate displays no effect and thereby proves that the observed optical response is indeed caused by the optical response of BiVO_4_ (Figure 1f bottom panel). Repeated switching of the illumination also shows that the force response is reversible and fast, both upon turning on and upon turning off the illumination. The off response is much faster than reported before for the relaxation of photoinduced charges on BiVO_4_ particles and Au-decorated TiO_2_ surfaces in inert gas. In those experiments, trapping of charge carriers in long-living surface states led to lifetimes of minutes or days.^27−29,44,45^ We conclude from our experiments that the presence of the ambient electrolyte enables much faster relaxation by providing additional relaxation channels, e.g., by charge transfer to the liquid.

**Figure 2:**
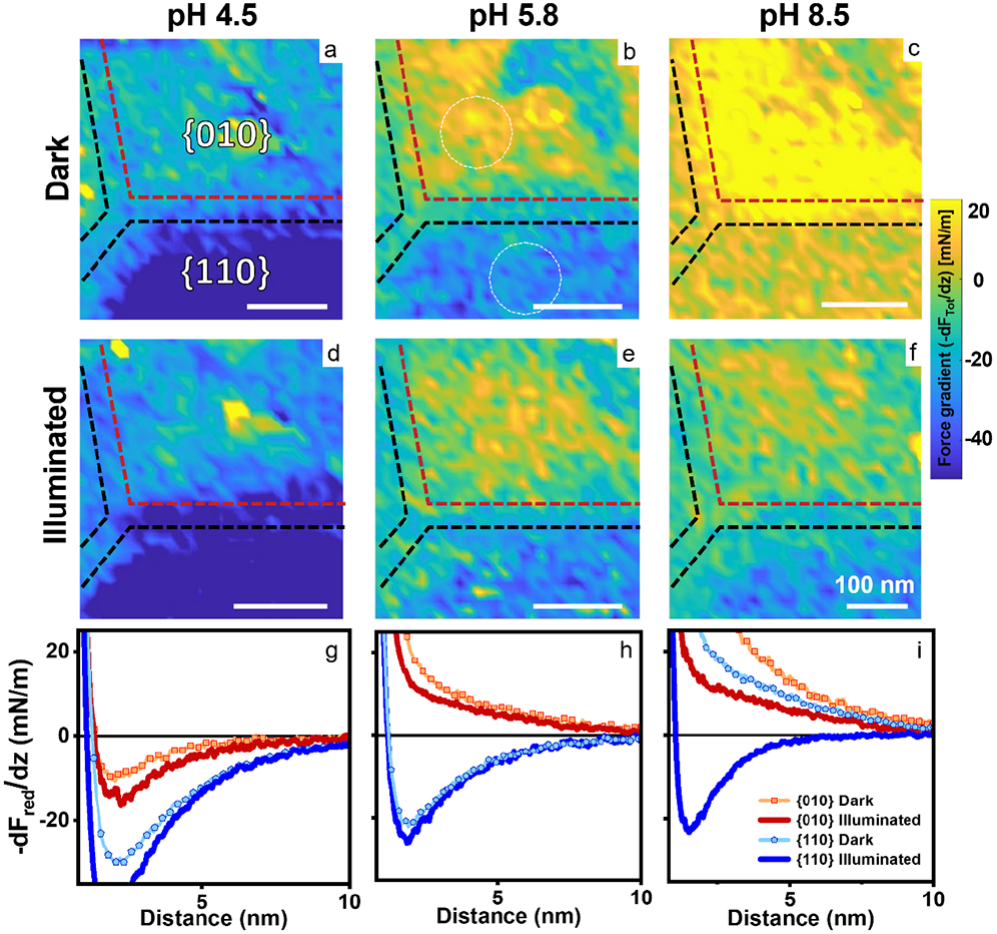
Facet-resolved photoresponse of an individual BiVO_4_ NP at variable pH. a) – f): force gradient (−dF_Tot_/dz) maps at 2.5 nm above the BiVO_4_ surface under dark and illumination conditions and pH values as indicated (NaCl concentration: 10 mM). g)–i): reduced force gradient (−dF_red_/dz) curves with and without illumination for both facets. Note that panels a–f show the total force gradient as measured, whereas panels g–i show the reduced force gradient after subtraction of the van der Waals attraction (*k*_Tot_–*k*_vdW_). All force maps are recorded on the same BiVO_4_ particle with the same probe with tip radius = 14.5 ± 2 nm. Corresponding NP topography images are shown in Figure S5.

To further explore the effect of the electrolyte composition, we analyze the force curves obtained for an individual BiVO_4_ NP at pH values of 4.5, 5.8, and 8.5 with and without illumination, as shown in Figure 2. First, we find that the averaged electrostatic forces are attractive (blue colors) at low pH and become increasingly repulsive (yellow) with increasing pH, corresponding to a transition from a positive surface charge at low pH to a negative one at high pH for both facets. This is qualitatively expected for oxide surfaces, which generally have the capability of pH-dependent (de)protonation of hydroxyl surface sites. Second, it is also immediately apparent from the force gradient maps that the charge on the {010} facet is always more repulsive (or less attractive) than on the {110} facet. Moreover, the {010} facet reverses sign at a lower pH corresponding to a lower isoelectric point *(IEP)* on the basal plane compared with the side facets. From the very small surface charge densities and potentials of the {010} and the {110} facets at pH 4.5 and 5.6, respectively, we infer that the corresponding facet-specific *IEPs* will be close to these values. The observed difference in facet-specific *IEPs* is qualitatively plausible given the higher density of *O*^2–^ lattice sites according to crystallography.^42,46,47^ The third important observation from Figure 2 is that illumination always makes the tip–sample interaction less repulsive (or more attractive) for all facets and pH values investigated. This corresponds to a universal accumulation of positive photoinduced charge carriers on both facets at all pH values. At pH 8.5, this leads to a photoinduced reversal from repulsive to attractive forces on the {110} facets, corresponding to a reversal of the sign of the charge density (Figure 2f).

**Figure 3:**
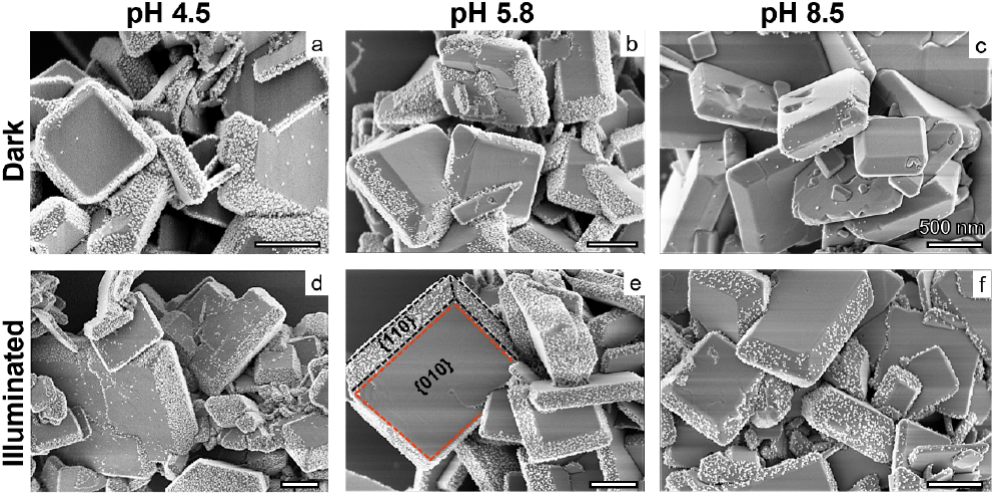
SEM images of BiVO_4_ particles after colloidal adsorption of 10 nm silica particles, at different pH (10 mM NaCl) with and without illumination. At pH 4.5 and 5.8, negatively charged SiO_2_ nanoparticles are adsorbed only on positively charged {010} facets. At pH 8.5, in the dark, the negatively charged facets and silica nanoparticles repel each other; thus, no adsorption of SiO_2_ particles is observed. Note the light-induced deposition of silica NP on the {110} facets at pH 8.5, which is absent in the dark. Note also the selective decoration of defects on the {010} facets, in particular at pH 4.5.

To probe the macroscopic consequences of the pH- and facet-dependent local surface charge, we perform a colloidal heteroaggregation test by adding negatively charged silica NPs to the suspension of the BiVO_4_ NPs analogue to our earlier measurements on SrTiO_3_^17^. Consistent with the higher *IEP*, adsorption of the negatively charged silica NPs is always more pronounced on the trapezoidal {110} facets compared to basal planes (Figure 3). On the basal planes, electrostatic repulsion prevents silica adsorption for all conditions investigated. In contrast, for the {110} facets, adsorption is only suppressed at the highest pH in the absence of illumination. Upon illumination, however, the initially negatively charged {110} facets at pH 8.5 turn positive and hence attract silica (Figure 3f) consistent with the observations in Figure 2.

**Figure 4:**
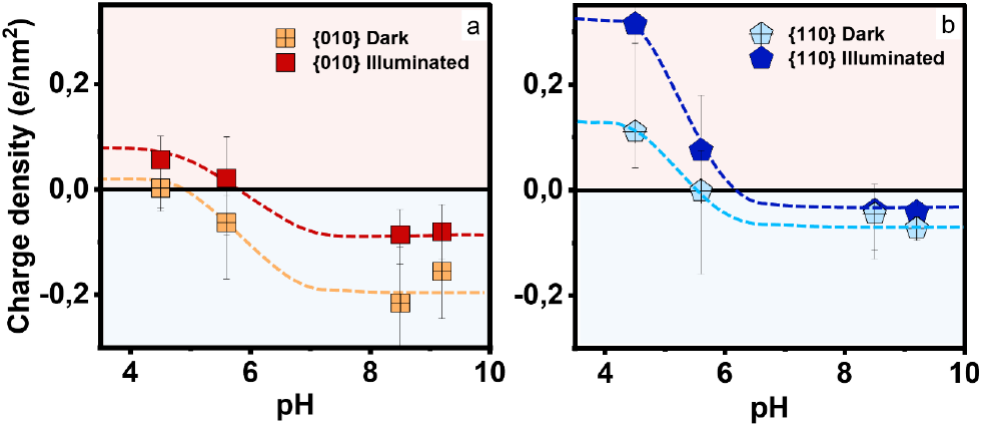
Local surface charge density of {010} (a) and {110} (b) facets of BiVO_4_ NPs in the dark (open symbols) and under illumination (filled symbols) in 10 mM NaCl solution. Error bars: statistical standard deviations from 6 to 10 independent measurements. Dashed lines are guides to the eye. The corresponding point of zero electrokinetic mobility from light scattering measurements of NP suspensions is ≈3 (see Figure S6b).

AFM measurements of many NPs in separate experiments confirm the trends shown in Figure 2. Averaged facet-selective titration curves show that both facets switch from a positive surface charge at low pH to a negative one at higher pH, Figures 4 and S6. The corresponding facet-dependent isoelectric points are *IEP*_{010}_ ≈ 4.5 and *IEP*_{110}_ ≈ 6. Both values are substantially higher than the commonly reported “isoelectric point” of BiVO_4_ of ≈3,^10,48^ which is off the scale of Figure 4. This low value, however, which we also confirm for our particles (see Figure S6b), is deduced from electrokinetic ζ-potential measurements by using the incorrect assumption of spherical particles with a homogeneous surface charge. The underlying analysis neglects anisotropy, facet-dependence, and—importantly—the effect of local surface defects, in particular along the edges of the particles (see below). This value should therefore be denoted as a point of zero electrokinetic mobility, *PZEM*, rather than an isoelectric point.^17^ Local surface charge densities on different facets thus do not necessarily vanish at the corresponding pH, as is apparent from Figures 2 and 4. This implies that the electrostatic interaction of charged solutes such as precursor ions during photodeposition or impregnation, organic molecules, and dyes vanishes at best for certain parts of the NP surface even if the pH of the solution corresponds to the *PZEM*.

The averaged surface charge data in Figure 4 also confirm that both facets become more positively (or less negatively) charged upon illumination, leading to an overall upward shift in both facet-specific titration curves. This result is consistent with the band structure of BiVO_4_: as an n-doped semiconductor with an intrinsic upward band bending at the surface, it is indeed expected that photogenerated holes are attracted toward the surface and photogenerated electrons are repelled.^6,28,29^ Conversion of the surface charge densities to local surface potentials using Grahame’s equation leads to values ranging from ∼ – 100 to +100 mV (Figure S6c,d). The photoresponse depends strongly on the specific facet and on the ambient pH with a maximum response of ∼ + 55 mV^a^ (Figure S6). Both, the absolute values of the surface potentials and the photoresponse, are substantially higher than typically reported for pristine (i.e., noncocatalyst-functionalized) BiVO_4_ in air^27−29^ (see Figure S7). Without electrolyte, comparable surface photovoltages of several tens of mV on BiVO_4_ are only observed in the presence of cocatalysts.^29^ Hence, we conclude that the interaction with the electrolyte can enhance the efficiency of the electron–hole pair separation to an extent that is—for favorable pH values—comparable to the effect of cocatalysts.

### Local Surface Charge and Photoresponse of Surface Defects

**Figure 5:**
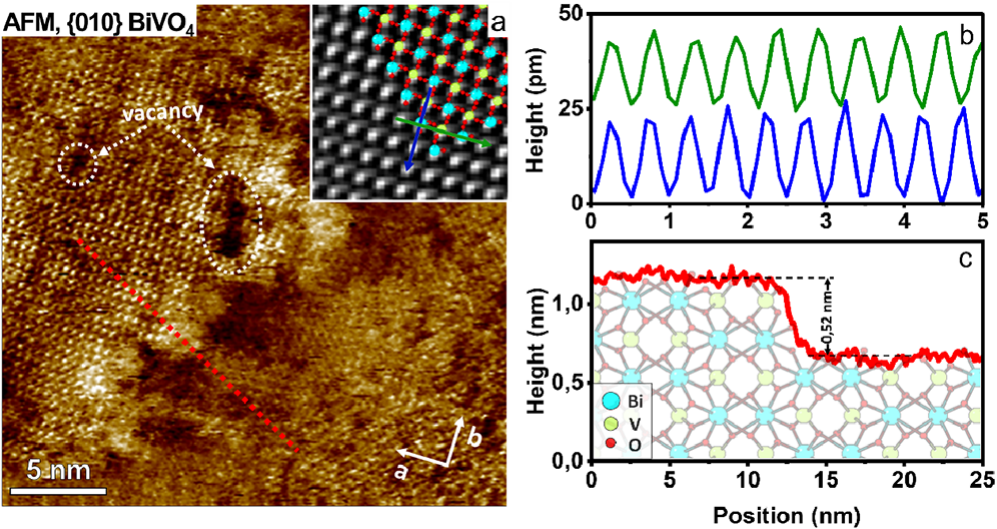
Atomic resolution imaging on BiVO_4_ nanoparticles in ambient electrolyte. a) High-resolution phase image on the {010} facet on BiVO_4_ measured in 100 mM NaCl at pH 6. It displays a rectangular lattice structure on {010} of BiVO_4_ with lattice parameters *a* = 0.497 nm and *b* = 0.524 nm and regions with disordered nonperiodic structure, vacancy defects, and a unit cell step. Top insets represent zoomed and Fourier-filtered view of atomic scale images with the superimposed X-ray resolved structure of {010} BiVO_4_ with unit cell parameters *a* = 0.5084 nm, *b* = 0.5214 nm, and *c* = *b* = 0.5214. AFM-resolved protrusions agree well with the X-ray crystal structure and arrangement of atoms. b) Height profiles in the blue and green directions shown in the bottom inset of (a) display periodicities of approximately 0.497 and 0.524 nm. c) Height profile taken along the blue line in (a) shows the unit cell step.

**Figure 6:**
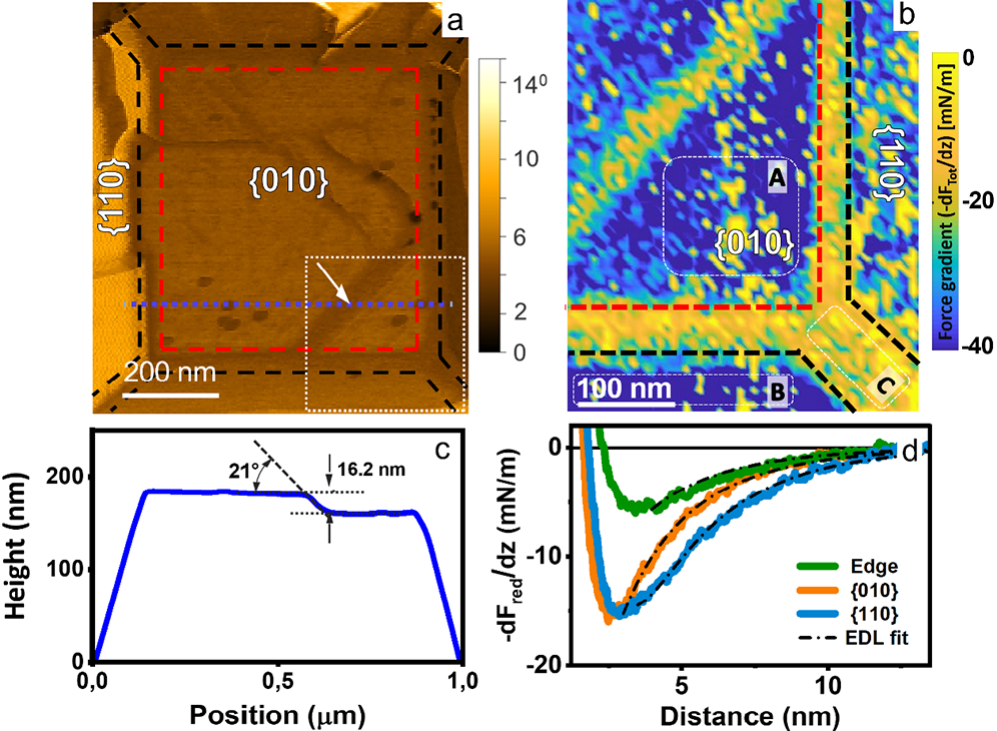
Influence of surface defects on local surface charge density at pH 4.8. a) AFM phase image showing various defects on the {010} basal plane. b) Total force gradient map at 2.5 nm above the BiVO_4_ surface taken in bottom right corner of panel a) (tip radius = 22.14 ± 2 nm). Note the pronounced repulsive forces at the microfacet (arrow in a)) and along the edges between adjacent facets. c) Height profile along dashed line in (a). d) Reduced force gradient (*k*_red_ = −dF_red_/dz = *k*_Tot_ – *k*_vdW_) vs distance corresponding to positions A, B, and C in panel (b). AFM topography images corresponding to panel (a) and 2D force map (panel b) are shown in Figure S8.

**Figure 7:**
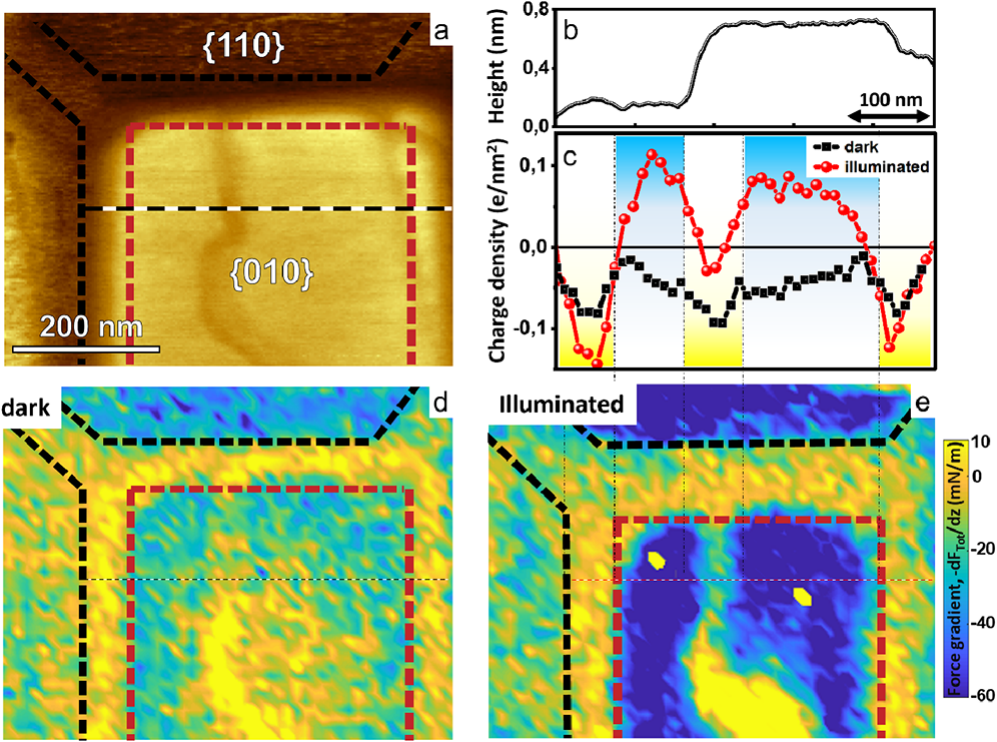
Photoresponse of a BiVO_4_ NP with a surface defect at pH 5.6. a) AFM Phase image of NP with a defect corresponding to a single unit cell step on the {010} plane. b) Height profile along the dashed line in (a). c) Local charge density along dashed lines in (d) and (e) without (black) and with (red) illumination. d),e) force gradient maps without and with illumination, respectively. Corresponding AFM topography images and an additional charge profile are shown in Figure S9.

Notwithstanding these consistent trends, we also observe substantial variations between different NPs as reflected in the large statistical error bars in Figure 4. What are the origin and consequences of the large particle-to-particle variations in our system? This could be related to variability in particle size, facets ratio, and surface defects that have a significant impact on photoinduced charge separation and overall photocatalytic activity.^28,49,23^ Yet, generally, it is also well-known that BiVO_4_ as a material is less stable and perfect than other photocatalytically active materials such as SrTiO_3_. This is manifested in a higher susceptibility to corrosion and a poorer crystallinity and morphology of as-synthesized BiVO_4_ NPs.^11,16,20,42,50^ In our AFM measurements, we routinely found surface defects on our BiVO_4_ NPs. Focusing first on flat regions in the center of the {010} facet of the BiVO_4_ particle, we find extended regions with a width of a few tens of nanometers where we can image the ideal atomic lattice of the material. Atomically resolved AFM images show uniformly spaced protrusions in a rectangular structure with spacings of approximately 0.50 and 0.52 nm in the *a* and *b* directions. These values are consistent with the size of the bulk truncated crystallographic surface unit cell^46,47^ of the {010} facet of BiVO_4_ and thus display no indications of surface reconstruction, Figure 5a. Next to regions of perfect crystallinity, we routinely observe extended regions of various types of defects, including vacancies of one or few atoms, more extended vacancy islands, unit cell steps, small microfacets, and disordered transition regions between adjacent facets with a width up to several tens of nanometers (Figures 5–7). The microscopic structure of defects shown in Figure 5 is somewhat rugged but remains constant throughout hours of observation, indicating the good overall stability of the material.

The nature and distribution of defect vary substantially from particle to particle. We believe that they are a major source of variability in the charge distribution and photoresponse in Figure 4. While the exact nature of defects is often difficult to identify, it is clear from the force response that the disordered regions between adjacent facets typically display a local excess negative charge (yellow in Figures 6b and 7d,e). This is consistent with earlier observations on defects on NPs of gibbsite^38^ as well as SrTiO_3_.^17^ For instance, we extract for edge region C in Figure 6b, σ_edge_ = +0.013 e/nm^2^ at pH 4.8, whereas the adjacent {010} and {110} facets display local charge densities of σ_{010}_ = +0.065 e/nm^2^ and σ_{110}_ = +0.118 e/nm^2^, respectively. We attribute this excess negative charge either to localized electronic states below the Fermi level or to hydroxyl groups at the defect site that become deprotonated.

Figure 7 shows a different NP at a somewhat higher pH of 5.6. The average charge density in homogeneous regions of the {010} facet is slightly negative. Similar as in Figure 6, the defect-rich regions at the border between adjacent facets display more repulsive forces and hence a more negative local surface charge density than the adjacent terraces. In addition, this specific particle displays a defect that runs across the {010} facet roughly from the top to bottom. A topographic cross section reveals a height of this defect is ≈0.53 nm (Figure 7b), which is close to a single unit cell along the [010] direction analogue to the one imaged at high resolution in Figure 5c.

Analyzing the force curves obtained with the blunter tip (as in panels a–d) along a line perpendicular to the step reveals that the local negative surface charge density is substantially enhanced along the defects (Figure 7c and Figure S9), as illustrated by the yellow color in the force gradient maps. The apparent width of the defect in both the topography image and the charge profile is ≈40 nm. We attribute this apparent width to a convolution of the actual microscopic step width (i.e., < 1 nm; see Figure 5) and the radius of the tip (≈27 nm for the present experiment, see Figure S1b. Since the defect is much narrower than the tip, it also seems reasonable to decompose the total electrostatic force into two contributions, one arising from the charge on the facets (weighted by the tip–sample interaction area) and a second one due to the interaction with the charges along the one-dimensional defect. Following this approach, we find that the step edge carries a negative charge density of approximately −1e/nm in the dark (see Supporting Information Note 1). Upon illumination, the force gradient on both the {010} basal plane and the {110} side facets becomes more attractive at pH 4.5 and 5.6, indicating the accumulation of positive photogenerated charge carriers on the facets, as discussed above (dark blue color Figures 7e and 4). On top of the step edge, however, the (averaged) charge density in Figure 7c increases less than on the facets. This implies that the step edge actually accumulates negative charge carriers. Following the decomposition described above, we find indeed that the one-dimensional charge density increases to approximately −2 e/nm. A similar accumulation of negative photoinduced charge carriers is also seen for the wider disordered regions at the edges between adjacent facets (Figures 7c and S9), which are believed to contain a higher density of broken bonds, similar to the step edge. We see two plausible explanations for this observation: first, the structural defects might involve localized electronic states within the gap that become occupied by photogenerated electrons.^22,23,51^ Alternatively, the photogenerated electrons might also change the configuration of chemical bonds at the surface and induce variations in the hydroxylation(hydration) and deprotonation of surface-bound OH groups.^6,52^ Based on our measurements, we cannot distinguish between microscopic scenarios that lead to the same change in local charge.

## Conclusions

In conclusion, our observations have important consequences for the separation of photogenerated charge carriers by facet and defect engineering. Commonly, it is assumed that photogenerated charge carriers in photocatalytic NPs are separated under the influence of the internal electric fields that are generated by the different surface potentials of adjacent facets.^9,34^ Our results demonstrate that (i) surface potentials and photoresponse are substantially enhanced by the interaction of the semiconductor with the ambient electrolyte. They depend strongly on the electrolyte composition, in particular on its pH; (ii) disordered regions at the boundary between adjacent facets as well as surface defects are omnipresent on particulate BiVO_4_ photocatalysts. Both display local surface potentials that are typically more negative than the adjacent facets. This affects the separation of photogenerated charge carriers and thereby induces an enhanced local photoresponse. Based on the observed frequency and the values of the local surface potentials, we conclude that surface defects are very likely to have an important influence on the macroscopic performance of BiVO_4_ NPs as photocatalysts. (iii) Another important aspect relates to the contrast in our images of the disordered regions and defects. It is clear from Figures 2, 6, and 7 that the lateral contrast between homogeneous facets and defects is rather sharp. Lateral variations of the surface potential are screened within, at most, a few tens of nanometers. This observed length scale is approximately hundred times smaller than the value recently proposed on the basis of optical fluorescence measurements.^35^ In fact, preliminary numerical calculations suggest that the true screening length should be dominated by the shortest screening length in the system, which is the Debye screening λ_D_ = 3 nm of the ambient electrolyte. Taking into account convolution effects with a finite tip size, our observations are compatible with this intrinsic screening by the electrolyte. This shortness of the screening length implies that electrical fields within the semiconductor are also more localized and stronger compared to a scenario with a semiconductor-controlled screening. This further contributes to the efficiency of charged defects in locally separating photogenerated charge carriers. Overall, our experiments show that the distribution of defects varies substantially between different BiVO_4_ NPs, and these variations have a strong influence on the local surface charges and hence most likely on the photocatalytic activity. Strategies to improve the control over crystallinity during the synthesis of BiVO_4_ may therefore be important to improve the performance and stability of the material.

## Experimental Section

### Synthesis of Faceted BiVO_4_ Nanoparticles

BiVO_4_ nanoparticles with anisotropic facets were synthesized as described in detail by Wang et al.^10^ Briefly, 36 mM Bi(NO3)_3_ were dissolved in 300 mL of a 2 M nitric acid solution. The pH of the solution was adjusted to 2 with ammonia solution (30 wt %) until the formation of the orange precipitate. After aging for 2 h, the precipitation was transferred to a 100 mL Teflon-lined stainless-steel autoclave and heated for 24 h at 200 °C. Then the powder was washed five times using ethanol and deionized water and finally dried for 12 h at 70 °C. A suspension of the powder (∼0.1 mg/mL) is prepared using deionized water (Millipore, Inc.).

### Sample Preparation

A 100 μL aliquot of this suspension is drop-cast onto freshly cleaned 12 mm × 12 mm transparent sapphire substrates. After a residence time of 5 min at 120 °C, in which the BiVO_4_ particles settle on the substrate, the sample is rinsed with deionized water to remove loosely bound particles and blown dry. The sapphire was cleaned in an ultrasonic bath for 10 min in a mixture of isopropanol, ethanol, and Millipore water (25/25/50% by volume) and subsequently rinsed with only Millipore water. Then, the substrate was air plasma cleaned (PDC-32G-2, Harrick Plasma, Ithaca, NY, USA) for 20 min. The surface coverage of BiVO_4_ nanoparticles on the substrate was less than 2–5%.

### Adsorption of SiO_2_ Nanoparticles onto BiVO_4_

100 μL of commercial SiO_2_ nanoparticles (LUDOX HS-30) with an average diameter of 12 nm were mixed with 2 mg of BiVO_4_ nanoparticles in 20 mL of a 10 mM NaCl solution (99% ACS reagent grade, Sigma-Aldrich). Afterward, the pH of the solutions was adjusted to 4.5, 5.8, and 8.5 by adding HCl (ACS reagent, 37%, Sigma-Aldrich) or NaOH solutions (ACS reagent, ≥97.0%, pellets, Sigma-Aldrich). All chemicals used were purchased from Sigma-Aldrich. To study the illumination effect, the suspension (BiVO_4_ with SiO_2_) was illuminated for 10 min using the 1.5G Xenon Lamp Sun simulator. After 10 min, the suspension that was kept in the dark and illuminated was centrifuged (3000 rpm for 15 min), and subsequently, the solution was drop-cast onto the substrate, blown dry, and imaged using SEM.

### Atomic Force Microscopy (AFM)

Dynamic amplitude modulation (AM-AFM) imaging and force spectroscopy measurements^17,37,38,53^ were performed with a commercial Asylum Research Cypher ES equipped with photothermal excitation. First, the topography of the sample under liquid was taken using AM-AFM imaging. From a large image, a suitable BiVO_4_ particle for force spectroscopy was chosen. Then, small amplitude (*A* ≤ 1 nm) AM-AFM force spectroscopy was performed on BiVO_4_ particles. The deflection (*u*), amplitude (*A*), phase (φ), and drive frequency (ω) are measured as a function of tip–substrate distance either on a fixed point on the particle surface (≈ 100 approach curves) or on a 2D grid over the area of interest using the built-in 3D force volume mapping of the Cypher AFM software. This results in a 3D volume of data of the tip sample approach and retraction curves. The tip–sample force gradient (interaction stiffness *k*_int_) was calculated from the amplitude and phase shift vs distance curves using standard force inversion procedures as extensively described by Liu et al. and Klaassen et al.^38,39^

Force spectroscopy measurements were performed using rectangular silicon cantilevers with conical silicon probe tips (MikroMash NSC36/Cr–Au BS) covered by a 1–2 nm thick native oxide layer. The cantilever stiffness *k*_c_, quality factor *Q*, and resonance frequency *f*_0_ are extracted from the thermal noise spectrum of the undriven cantilever in liquid at a distance of *h* = 500 nm from the substrate, where the tip–sample interaction is negligible. Typical values are *k*_c_ ∼ 1 N/m, *f*_0_ ∼ 25 kHz, and *Q* ∼ 3. To measure the tip–sample interactions, the cantilever was driven at a fixed frequency (*f* ≈ 0.97•*f*_0_) by an intensity-modulated blue laser diode that was focused on the gold coated topside of the cantilever close to its base. To protect tip sharpness, the amplitude signal was not allowed to drop below 80% of its free oscillation amplitude (*A*_0_ < 1 nm). The radius of the tip was determined after AFM data collection from scanning electron microscopy (SEM) images (see Figure S1). Atomic resolution imaging of the solid electrolyte interface^37,40,54^ was performed with ultrasharp Arrow UHF-AUD tips (Nanoworld, Neuchatel, Switzerland); *k* = 3.35 N/m, *f*_0_ = 600–1000 kHz, and *Q* = 11, tip radius ∼1 nm). The AM-AFM mode is operated with a free amplitude of *A*_0_ ≤ 0.3 nm, a high scan rate of ≈12 Hz, and an amplitude set point as high as possible, typically *A*/*A*_0_ ≥ 0.9. Prior to use, AFM tips were cleaned in a mixture of isopropanol and ethanol and subsequently to air plasma (PDC-32G-2, Harrick Plasma) cleaning for 15–30 min. The experiments were performed in a closed cell that allows liquid exchange and temperature control, *T* = 22.7 ± 0.5 °C. The cantilever was immersed in a droplet of liquid (0.2–0.4 mL) that was sandwiched between the substrate (1.2 × 1.2 cm^2^) and the top of the cell. The fluid was exchanged by using two syringes by injecting a new solution while completely removing the old solution. The liquid exchange was done by replacing at least 25 times the drop volume. Measurements were performed after an equilibration time of 15 min. The experiments are conducted in 10 mM NaCl, the optimal salt concentration for surface charge density of the tip and electric double-layer forces decay length (Debye length, λ_D_ = 3 nm). Lower salt concentrations reduce surface charge density at the silica AFM tip,^37,38^ while higher concentrations decrease the strength and decay length of EDL forces, reaching the tip of sample separation (<1–1.5 nm), where it is difficult to decouple the total interaction force into distinct contributions like DLVO and non-DLVO forces like hydration.^39^

All experiments were performed with a custom-built bottom illumination AFM stage provided by Asylum Research that was used with the Cypher ES AFM (Oxford Instruments Asylum Research, Santa Barbara, CA USA). The stage contains a white LED that provides variable intensity (0–30 sun) white light (∼420–680 nm). Part of this light is absorbed within the NPs before it reaches the surface. The light intensity at BiVO_4_ NP-electrolyte interfaces is approximately 1–2 sun.

### Surface Charge Determination from Force–Distance Curves

As described earlier,^17,37−39,41^ to obtain the surface charge densities, the experimental force–separation curves were fitted with theoretical DLVO force curves that have contributions from the electrostatic (*F*_EL_) or electric double layer (*F*_EDL_) interaction and van der Waals interaction *F*_VDW_
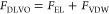


The electrostatic part was obtained by solving the full Poisson–Boltzmann equation with a boundary condition that involves a constant regulation^38,41,55^ (for details, see Supporting Information Note 2).

For the correction of the orientation of BiVO_4_ facets {010} and {110} with respect to the surface normal on absolute interaction forces and surface charge densities, see Su et al.^17^ and Supporting Information Note 3.

## Supporting Information Available

AFM topography images of all-characterized BiVO_4_ NPs, surface potential of {110} and {010} facets of BiVO_4_ NPs in the dark and under illumination; surface photoresponse of {010} and {110} facets of BiVO_4_ NPs in variable electrolyte; extraction of local surface charges from AFM force measurements using DLVO theory and charge regulation; and correction for the angle between the AFM tip and the facets of BiVO_4_ NPs in the force data (PDF)

## Footnotes

a We note that the photo response extracted from our data describes the light-induced variation of the potential drop across the diffuse part of the electric double layer. If the illumination induces substantial rearrangements in the condensed part of the electric double layer, this value may deviate from the actual surface photo voltage, i.e. the variation of the potential exactly at the solid-electrolyte surface.
