# Supplementary material for: Nanometer-Resolved Operando Photo-Response of Faceted BiVO4 Semiconductor Nanoparticles
Source: J Am Chem Soc. 2024 Jan 12;146(3):2248–56. doi: 10.1021/jacs.3c12666 (PMC10811660; doi:10.1021/jacs.3c12666)
Supplement: Supplementary file 1 — ja3c12666_si_001.pdf [file ja3c12666_si_001.pdf]

Supporting Information

Nanometer-resolved Operando Photo-Response of Faceted $BiVO_4$ Semiconductor Nanoparticles

Shaoqiang Su[1‡], Igor Siretanu[1‡], Dirk van den Ende[1], Bastian Mei[2], Guido Mul[2], Frieder Mugele[1]

[1]*Physics of Complex Fluids Group and MESA+ Institute, Faculty of Science and Technology, University of Twente, P.O. Box 217, 7500 AE Enschede, The Netherlands.*

[2]*Photocatalytic Synthesis Group and MESA+ Institute, Faculty of Science and Technology, University of Twente, P.O. Box 217, 7500 AE Enschede, The Netherlands.*

[‡]These authors contributed equally to this work.

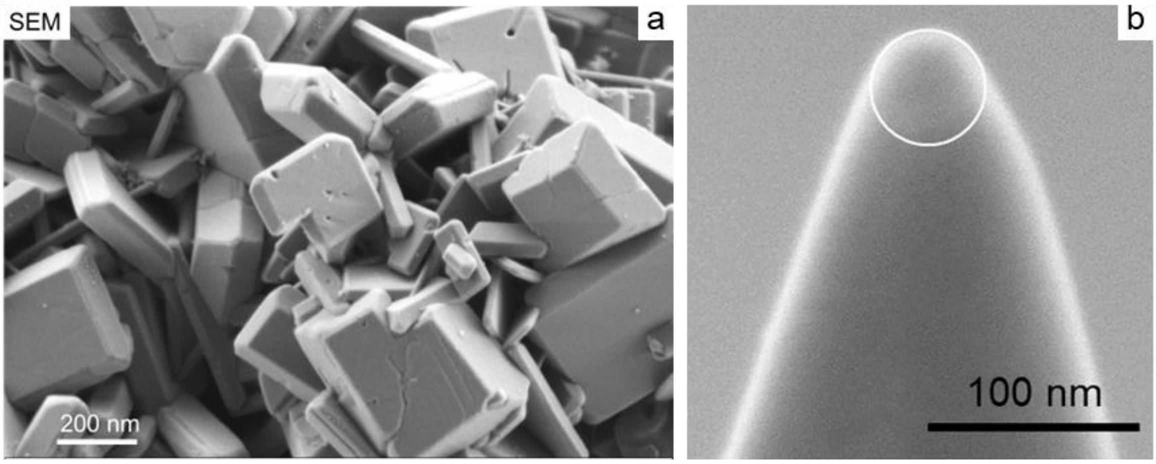

Figure S1. (a) SEM image of $BiVO_4$ nanoparticles on a silicon wafer. (b) Typical SEM image of AFM tip after experiment. SEM image of AFM tip (MikroMash NSC36/Cr-Au BS) used for AFM spectroscopy measurements shown in Figure 7. The radius of the AFM tip (R_{tip}=26.95) is obtained by fitting a circle to the hemispherical tip.

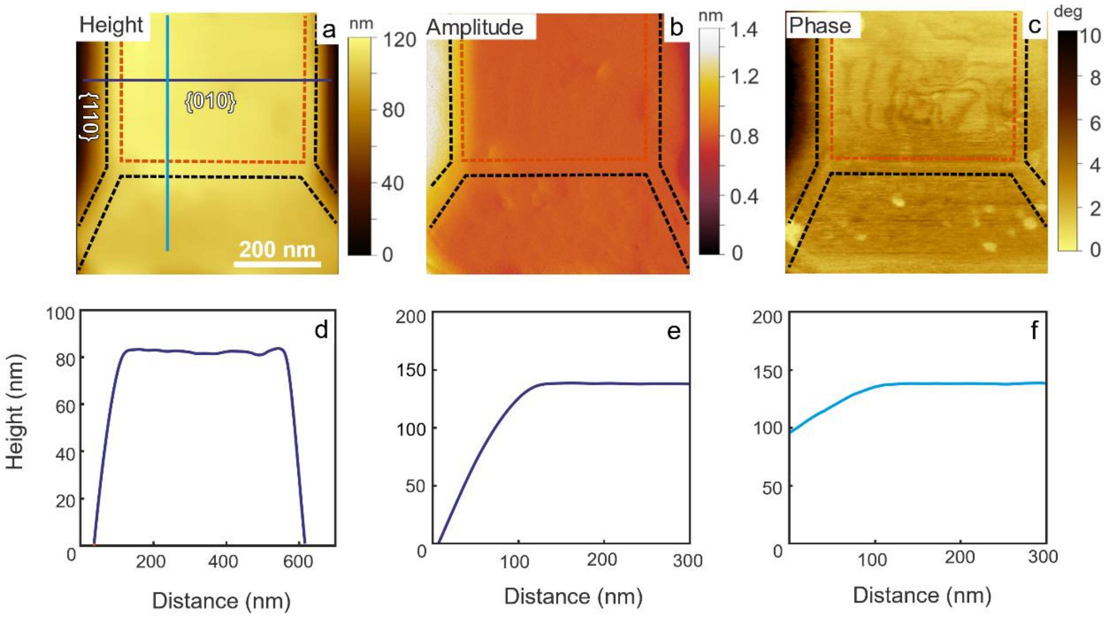

Figure S2. Amplitude modulation - AFM topography (a), amplitude (b) and phase (c) images of $BiVO_4$ nanoparticle adsorbed on sapphire in a 10 mM NaCl, pH 5.6 solution. These images correspond to the location where 2D-force map shown in Figure 1 c is collected. d)-f) height profile along solid lines in a). AFM tip parameters: MikroMash NSC36 with silicon tip and cantilever: Q factor = 3.7; resonance frequency = 31.65 kHz; spring constant = 1.3 N m^{-1}; tip radius = 26.9 ± 2 nm).

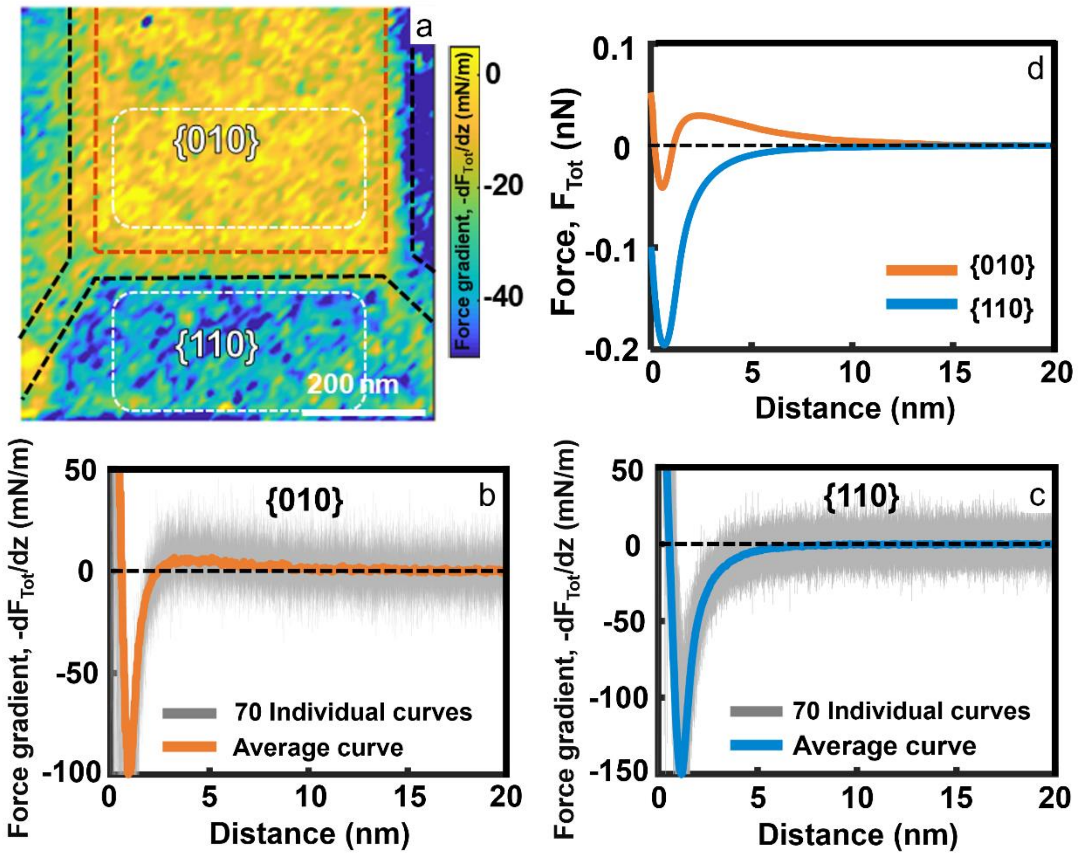

Figure S3. (a) Color-coded 2D total interaction stiffness (k_{TOT}) map or total force gradient ($-dF_{Tot}/dz$) map shows tip–$BiVO_4$ NP nanoparticle interactions in 10 mM NaCl solution at pH = 5.8. The 2D map was extracted from a 3D force versus distance (FD) volume plot when the tip is 2.5 nm away from the

$BiVO_4$ particle surface. The 3D force versus distance (FD) volume plot was obtained by measuring 60 × 60 tip-$BiVO_4$ interaction curves. The lateral distance between each force–distance curve is 10 nm. Colour code: yellow and blue colours indicate repulsive and attractive interactions, respectively. The topography image corresponding to the force map is shown in Figure S2 (Supporting Information). b) and c) panels display individual and average total force gradient versus distance curves across a flat region at the center of {010} (b) and {110} (c) facets of $BiVO_4$ particles (marked with white rectangles on the 2D map in (a)). Shaded gray regions are the 70 individual tip sample force gradient approaches. Thick orange {010} and blue {110} lines are their respective averages. d) average force–distance curves obtained from the force gradient data by numerical integration.

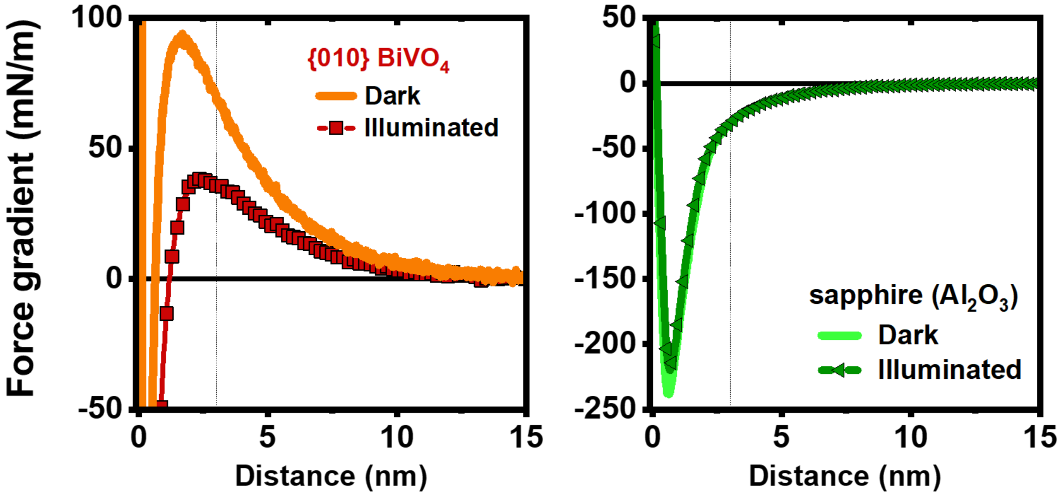

Figure S4. Average force gradient versus distance curves obtained on one single point of {010} facet of $BiVO_4$ and sapphire (Al_2O_3) substrate during the one round of illumination and in dark. The average is for forces recorded during 8 minutes at 1 Hz ramp rate. Illumination reduces the electrostatic repulsion on the {010} facet while the sapphire substrate control surface displays no light response. AFM tip parameters: MikroMash NSC36 with silicon tip and cantilever: Q factor = 4.1; resonance frequency = 30.163 kHz; spring constant = 1.22 N m^{-1}; tip radius = 22.5 ± 2 nm).

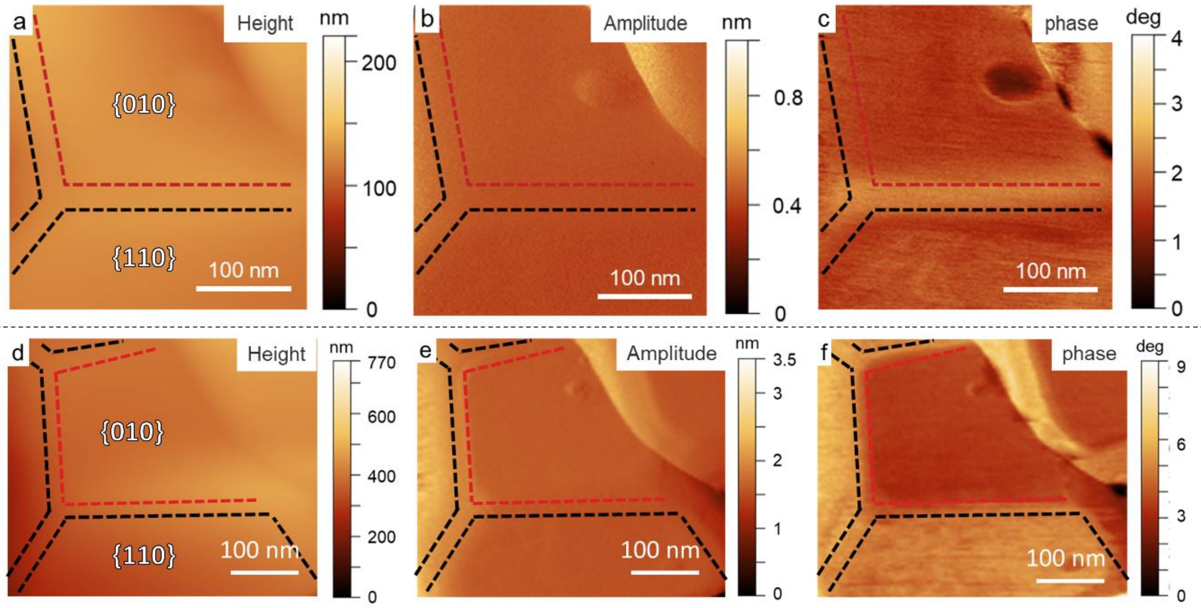

Figure S5. AM-AFM topography, amplitude, and phase images of $BiVO_4$ nanoparticle adsorbed on sapphire in 10 mM NaCl, pH 5.6. These images correspond to the location where the force maps shown in Fig. 2 are collected. (a, b, and c) correspond to images before the first 3D force map is recorded, while (d, e, and f) are images of the $BiVO_4$ particle after completion of the 3D force maps (at pH 4, 6, and 9 with 10 mM NaCl, in the dark and under illumination), as shown in Figure 2. The data indicates that the $BiVO_4$ nanoparticle did not change (dissolve or degrade) over the course of all experiments. The order of the experiments was as follows: pH 5.6, 10 mM NaCl (dark); pH 8.5, 10 mM NaCl (dark); pH 4.5, 10 mM NaCl (dark); pH 4.5, 10 mM NaCl (illuminated); pH 5.6, 10 mM NaCl (illuminated); and pH 8.5, 10 mM NaCl (illuminated). Tip parameters: Q factor= 3; resonance frequency= 19.110 kHz; spring constant = 0.65 N m^{-1}; tip radius = 14.5 nm ± 2 nm.

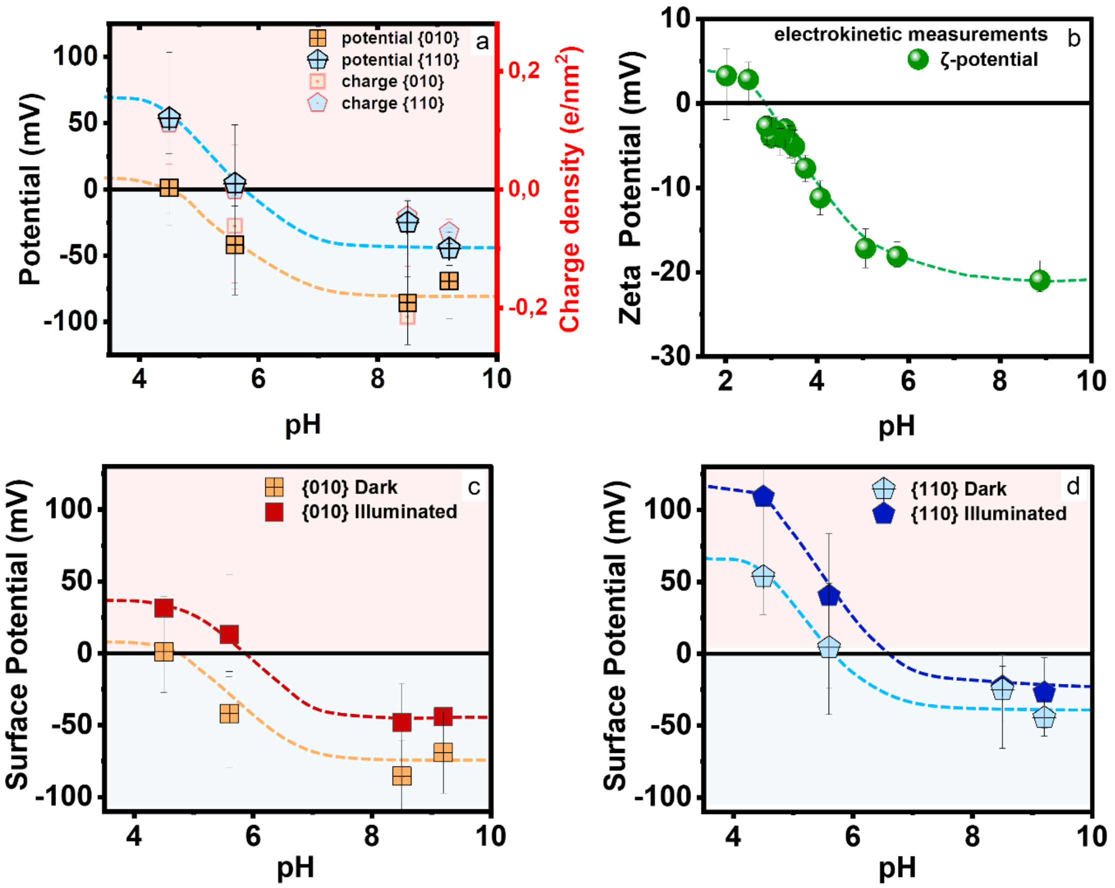

Figure S6. Electrical (potential) properties of {110} and {010} facets $BiVO_4$ nanoparticles a) Average diffuse layer potential (left Y axis) and surface charge (left Y axis) of {010} and {110} facets of $BiVO_4$ nanoparticle as a function of pH in 10 mM NaCl with and without illumination. Data reveal facet-dependent isoelectric points of $IEP_{\{010\}} \approx 4.5$ and $IEP_{\{110\}} \approx 6$. b) Measured zeta potential of $BiVO_4$ nanoparticle suspension as a function of pH in 10 mM NaCl. Error bars are statistical standard deviations from 3 separate measurements (green dash line is to guide the eyes). Suspensions of our $BiVO_4$ NPs display an (average) IEP of pH 3 in electrokinetic measurements of the ζ-potential. Average local diffuse layer surface potential as a function of pH in 10 mM NaCl with and without illumination for {010} facet (panel c) and {110} facet (panel d). Error bars are statistical standard deviations from 6-10 independent measurements per condition. The thick, dashed lines are a guide for the eye. Surface potential values are converted from the surface charge values in Figure 4 using the Graham equation.

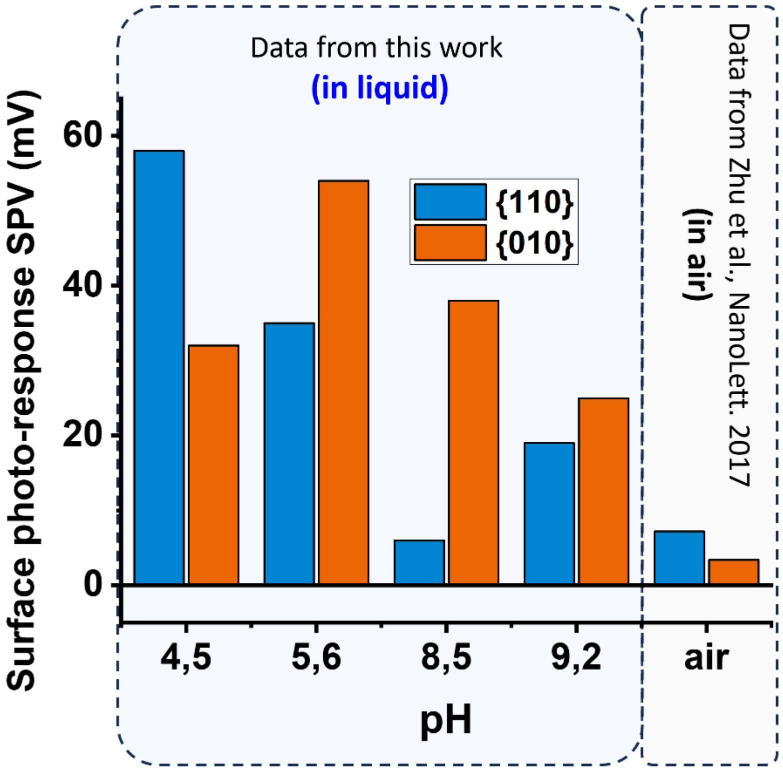

Figure S7. Surface photo-response of {010} and {110} facets of $BiVO_4$ NPs in electrolyte of variable pH. Left side of the graph are data extracted from Figure 4 and Figure S6 c and d. The photoresponse depends strongly on the specific facet and on the ambient pH. Right part of the graph: surface (photo)voltage (SPV) measured in air for {010} and {011} facets of $BiVO_4$ (data from Zhu et al., NanoLett. 2017[1]).

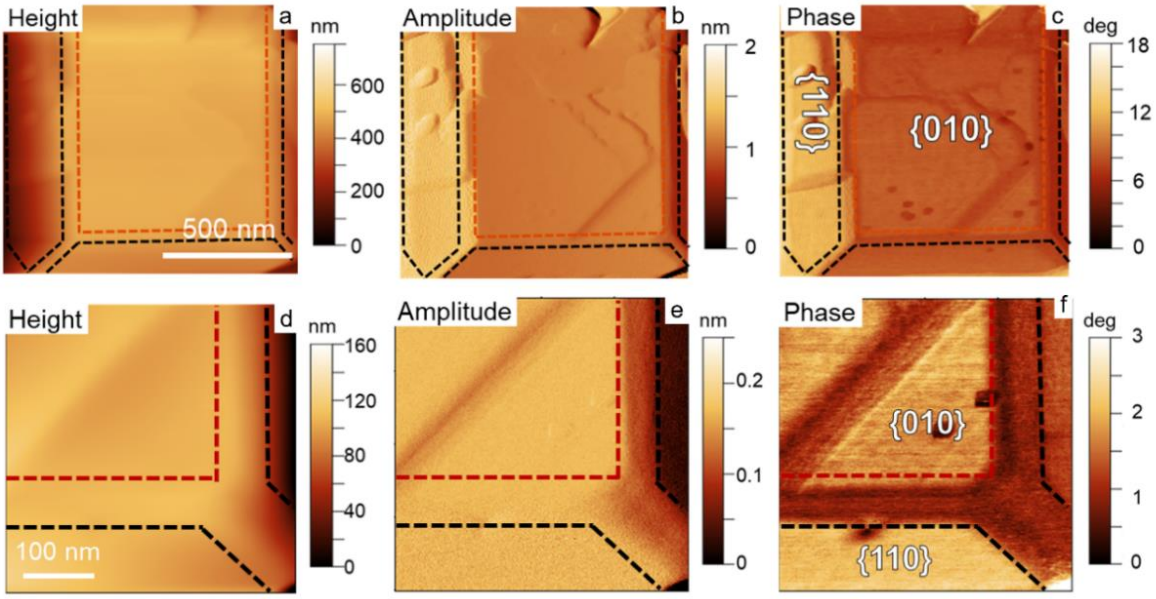

Figure S8. AFM images a) Height; b) Amplitude; c) Phase of the $BiVO_4$ nanoparticle shown in Figure 6a. AFM images d) Height; e) Amplitude; f) Phase corresponding to the location where the two-dimensional 2D force map measurement shown in Figure 6b is performed. AFM tip parameters used to acquire AFM images and the force map shown in Figure 6b: MikroMash NSC36 with silicon tip: Q factor = 3.7; resonance frequency = 31.65 kHz; spring constant =1.3 N m^{-1}; tip radius = 22.14 ± 2 nm.

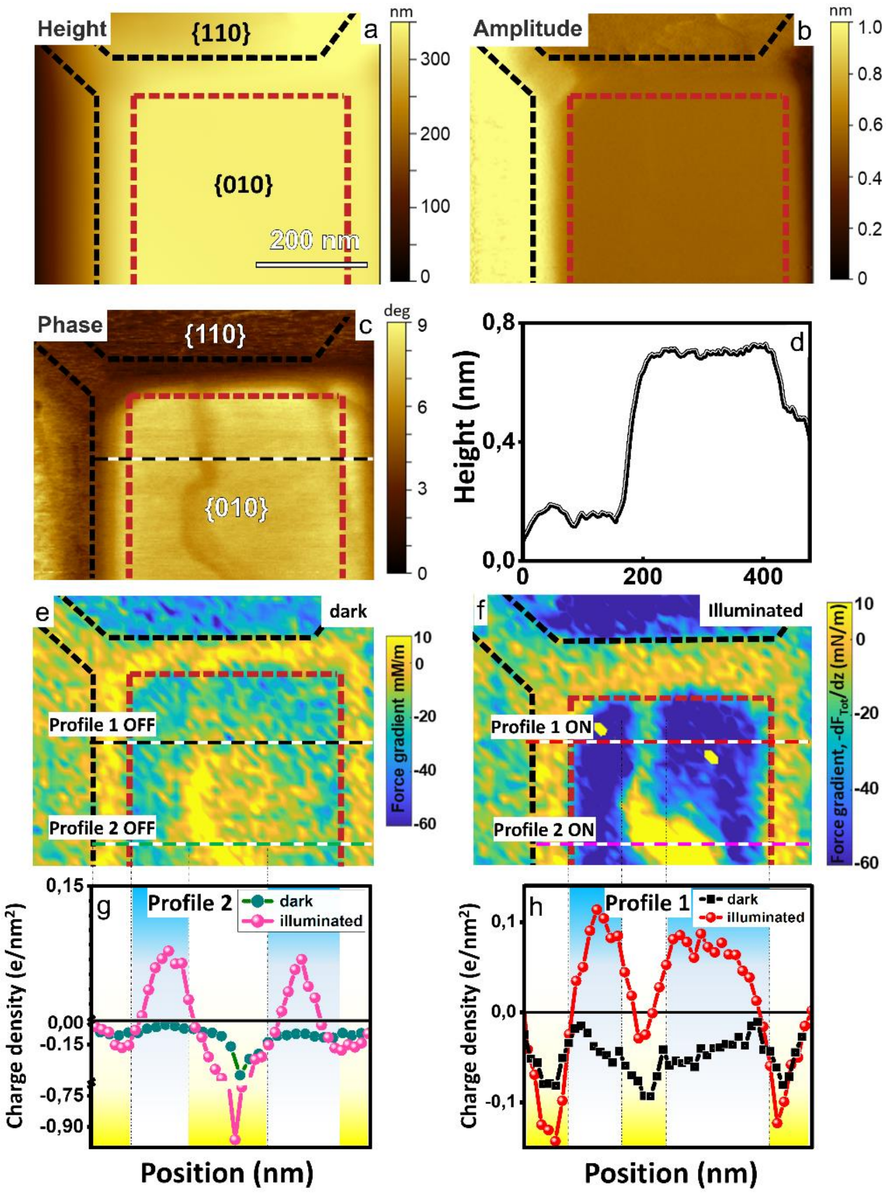

Figure S9. AFM images a) Height; b) Amplitude; c) Phase of $BiVO_4$ nanoparticle shown in Figure 7a. AFM images correspond to the location where two dimension (2D) force maps measurement shown in Figure 7d and e are performed. d) height profile along dashed line in c). e) force gradient maps without and with illumination, respectively. g-h) local charge density along dashed lines (profile 1 and profile 2) in e), f) without (black and green charge profiles) and with (red and pink charge profiles) illumination.

Supplementary Information Note 1:

Estimate of line charge density

For a tip with a flat bottom with radius r_{2D}, the tip-sample interaction area is $A_{ts} = \pi r_{2D}^2$. The 2D diffuse layer charge density σ_{app} reported throughout this work is extracted based on the assumption that the charge density on the sample is homogeneous within the interaction area. Hence, the total charge detected by the tip is $Q_{tot} = A_{ts}\,\sigma_{app}$. Assuming that a unit cell step can be described as a sharp one-dimensional charge defect, we estimate that the total charge experienced by the tip on top of the defect is composed of two contributions, namely the one from the 1D-line charge density λ along the defect and the contribution from the adjacent terraces, i.e.

$$Q_{tot} = A_{ts}\,\sigma_{app} = A_{ts}\,\sigma_{facet} + 2\,r_{2D}\,\lambda \qquad (1)$$

Hence, we can estimate the line charge density as:

$$\lambda = \frac{A_{ts}}{2r_{2D}}\left(\sigma_{app} - \sigma_{facet}\right) = \frac{\pi\,r_{2D}}{2}\left(\sigma_{app} - \sigma_{facet}\right) \qquad (2)$$

From Fig. 7b and c, we can extract $\sigma_{app}^{dark} \approx -0.1\ e/nm^2$ right on top of the step and $\sigma_{facet}^{dark} \approx -0.05\ e/nm^2$ in the middle of the facet in the dark. Under illumination (red curve in Fig. 7c), we have $\sigma_{app}^{illum} \approx -0.03\ e/nm^2$ and $\sigma_{facet}^{illum} \approx +0.08\ e/nm^2$. The 3D tip radius in the present experiments is $R_{tip} = 27nm$. Taking r_{2D} and the radius of the part of the tip that is within one Debye screening length of the surface ($\lambda_{Debye} = 3\ nm$), we find:

$$r_{2D} = \sqrt{R_{tip}^2 - \left(R_{tip} - \lambda_{Debye}\right)^2} = 12.3\ nm \qquad (3)$$

Using $r_{2D} = 27\ nm$, this leads to estimated line charge densities of $\lambda_{dark} \approx -1\ e/nm$ and $\lambda_{illum} = -2.1\ e/nm$, corresponding to distances between of adjacent elementary charges of approximately 1nm and 0.5nm, respectively. I.e. the one dimensional line charge density becomes more negative upon illumination. The fact that the local minimum above the step shifts upward in Fig. 7c is thus a consequence of the accumulation of positive charge on the facets whereas the defect actually accumulates more electrons.

Supplementary Information Note 2

As extensively described earlier[2-7], the measured force-distance curves are converted to surface charge using DLVO theory and charge regulation model for tip and silica-$BiVO_4$ nanoparticle sample. To do so, we calculate hypothetical force-distance curves for given surface charge and regulation parameter and compare these curves with the measured curves using the surface charge and regulation parameter as fitting parameters. To determine the force between the tip and sample surface, we first calculate the disjoining pressure $\Pi(D)$ in the gap with height D between them. This pressure can be split in a contribution Π_{vdW} due to van der Waals interactions and an electrostatic double layer contribution Π_{EDL}. The force on the tip is calculated by integrating Π over the spherical tip with radius R_{tip}:

$$F_{int}(D) = \int_D^{\infty} k_{int}(D)dD = 2\pi R_{tip} \int_D^{\infty} [\Pi_{EDL}(D) + \Pi_{vdW}(D)]\, dD \qquad (4)$$

The Van der Waals contribution, between an AFM probe with radius R and a flat surface was calculated using:

$$\Pi_{vdW}(D) = -\frac{A_H}{6D^2} \qquad (5)$$

where A_H is the Hamaker constant, and D is the tip to surface distance. The Hamaker constants A_H are fixed to $0.65 \cdot 10^{-20}$ J for the (silica-water-silica; tip calibration system) and to $2.8624 \cdot 10^{-20}$ J for the ((silica-water-$BiVO_4$)) system.

The electrostatic double layer contribution contains the required information on the surface charge and was obtained by solving the full Poisson-Boltzmann equation with a boundary condition that involves a constant regulation[4, 8-10]. For a 1-1 electrolyte it is given by:

$$\Pi_{EDL}(D) = k_B T \sum_i \rho_{b,i} \left(\exp\left(-\frac{Z_i q_e \psi(z)}{k_B T} \right) - 1 \right) - \frac{1}{2} \epsilon\epsilon_r \left(\frac{\partial \psi}{\partial z} \right)^2 \qquad (6)$$

where $k_B, T, \epsilon, \rho_{b,i}, q_e, Z_i$ are Boltzmann constant, the temperature vacuum permittivity, bulk number density of i-th ionic species, elementary charge, valency of i-th ionic species and electrostatic potential, respectively c_∞ the bulk ion concentration and e the elementary charge; $\psi(z)$ is the electric potential for $0 < z < D$.

Calculation of the electric double layer contribution requires knowledge of the potential $\psi(D)$ in the electrolyte. Here the potential ψ and its derivative are obtained by solving the Poisson-Boltzmann equation (PB)[6]:

$$\frac{\partial^2 \psi}{\partial z^2} = \frac{-q_e}{\epsilon\epsilon_r} \sum_i Z_i\, \rho_{b,i} \exp\left(\frac{-Z_i q_e \psi(z)}{k_B T} \right) \qquad (7)$$

with the boundary condition

$$(\hat{n} \cdot \nabla\psi)_s = \frac{-\sigma(\psi_s)}{\epsilon\epsilon_r} \quad (8)$$

The subscript s denotes quantities calculated at the surface. Here $\hat{n}$ is a unit normal vector and σ is the surface charge density, which we describe here using a constant-regulation model,

$$\sigma = \alpha + \beta\psi_s \quad (9)$$

α and β are fit parameters which minimize the function

$$\chi = \frac{1}{\sqrt{\sum_i^N \left(F_{EDL}(D_i) - F_{EDL,exp}(D_i)\right)^2 / N}} \quad (10)$$

where $F_{EDL}(D_i)$ and $F_{EDL,exp}(D_i)$ are theoretically calculated and experimentally measured electrostatic forces, respectively, for a separation of D_i between two surfaces.

For an isolated solid-electrolyte interface, the equation 7 reduces to the Grahame equation, which relates diffuse layer charge density to diffuse layer potential:

$$\sigma = \sqrt{8c_\infty \varepsilon_0 \varepsilon_r k_B T} \sinh\left(\frac{Z_i \mathrm{q_e} \psi_s}{2\mathrm{k_B T}}\right) \quad (11)$$

The point of intersection between equation 6 and the Grahame equation gives the isolated surface charge and potential. Once the ψ_s is known, we can then quantify the charge regulation behavior using the regulation parameter, p, that can then be calculated as

$$p = \frac{C_D}{C_D + C_I} \quad (12)$$

where, C_D and C_I are the diffuse layer capacitance and inner layer capacitance, respectively.

$$C_D = \left(\frac{q_e^2 \epsilon\epsilon_r}{2k_B T}\right)^{1/2} \cdot \frac{\sum_i Z_i \rho_{b,i}\left(exp\left(-\frac{Z_i q_e \psi_s}{k_B T}\right) - 1\right)}{\left[\sum_i \rho_{b,i}\left(exp\left(\frac{-Z_i q_e \psi_s}{k_B T}\right) - 1\right)\right]^{1/2}} \quad (13)$$

and,

$$C_I = \frac{2q_e^2 I_\infty \kappa^{-1} \beta}{k_B T} \quad (14)$$

where κ^{-1} and I_∞ are the Debye length (λ_D) and bulk solution ionic strength

The Debye length is given by,

$$\lambda_D = \frac{1}{k} = \sqrt{\frac{\varepsilon_0 \varepsilon_r k_B T}{2{q_e}^2 I_\infty}} \quad (15)$$

where ε_0 is the vacuum permittivity of the medium, ε_r is the relative permittivity, q_e is the elementary charge and I_∞ is the ionic strength, defined by $I_\infty = \sum_i Z_i^2 n_{i,\infty}$, with$Z_i$ and n_i being the valency and the concentration of i-th ionic species.

Both diffuse layer potential and inner layer capacitance enter the regulation parameter which represents a description of an isolated surface. The advantage of this parameterization is that the regulation parameter takes simple values for constant potential (CP) boundary conditions $p = 0$ and constant charge boundary (CC) conditions $p = 1$. Also, the model force distance curves that include the charge regulation (CR) boundary condition describe the experimental data of a significantly wider range than the approximate solutions for constant potential (CP) and constant charge (CC) solutions[4, 5]. The force is obtained by the integration of the pressure profile and by using the Derjaguin approximation. The Derjaguin approximation is expected to be valid, since the tip size is larger than the Debye length[6]. The force *vs.* distance (or force gradient) curves calculated using the theoretical model depend on a number of parameters, including the radius of the AFM tip *R*, the Hamaker constant *A*, ion concentration c^∞, the surface charge/potential σ_d/Ψ_d and the regulation parameter p. When the experimental force profiles are fitted within a nonlinear least-squares procedure, the salt concentration, the Hamaker constant and tip radius are known and fixed the regulation parameter p and the diffuse layer charge/potential σ_d/Ψ_d are extracted by fitting the DLVO force profile to the experimental data at separation distances between 2 to 15 nm. This procedure ensures that the influence of short-range forces such as hydration forces are not included in the physical model and the dominance of noise at larger distances are reduced. Note that the values reported throughout this work include the intrinsic charge surface density (or local electrostatic potential) of the solid, possibly adsorbed ions in the Stern layer, and the charge density in the space charge region of the semiconductor.

Supplementary Information Note 3

To account for the influence of the angle between the tip and the individual facets of $BiVO_4$ or the tilt of a substrate on measuring the interaction force between the tip and the substrate, we consider the geometry as sketched in Figure S10. We are interested in the force $F_{ts} = |\vec{F}_{ts}|$ as a function of the tip-sample distance h_{ts}. However, with our AFM probe, we can measure only F_{ts}^{z} as a function of the vertical distance $z_{ts} = z_t - z_s$. Based on the depicted sketch (Figure S10) for horizontally oriented {010} facets, where the AFM tip is vertical to the sample surface, the $F_{ts} = F_{ts}^{z}$ and $h_{ts} = z_{ts}$. However, for a finite angle θ between the tip and the {110} facets, or in a more general situation, if the sample surface is inclined with an angle θ, we need to correct for the projection of the tip-sample distance and the interaction force onto the local surface normal. From the sketched geometry shown in Figure S10, we derive the relations between F_{ts}, h_{ts} and θ, namely sample tilt or angle between the tip and the sample:

$$h_{ts} = z_{ts} cos\theta, \qquad F_{ts} = \frac{F_z^{ts}}{cos\theta} \qquad (16)$$

Besides the force, often the interaction stiffness (k_{ts}) or force gradient $k_{ts} = -\frac{\partial F_{ts}}{\partial h_{ts}}$ is needed to analyze the data, which can be expressed as:

$$k_{ts} = \frac{k_{int}}{cos^2\theta} \text{ or } \frac{\partial F_{ts}}{\partial h_{ts}} = \frac{1}{cos^2\theta}\frac{\partial F_z^{ts}}{\partial z_{ts}} \qquad (17)$$

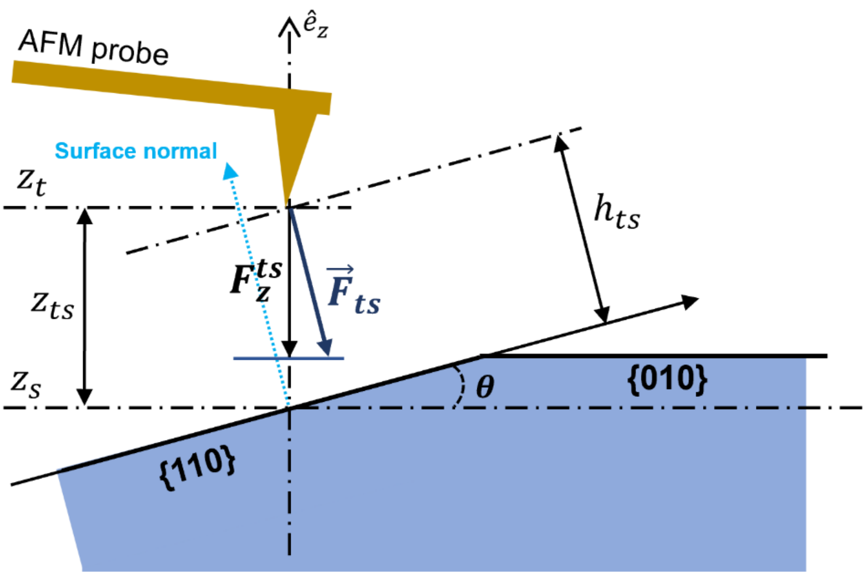

Figure S10. Tip-sample (faceted $BiVO_4$ particle) geometry.

References

(1) Zhu, J.; Pang, S.; Dittrich, T.; Gao, Y.; Nie, W.; Cui, J.; Chen, R.; An, H.; Fan, F.; Li, C. Visualizing the nano cocatalyst aligned electric fields on single photocatalyst particles. *Nano Letters* **2017**, *17* (11), 6735-6741.

(2) Klaassen, A.; Liu, F.; Mugele, F.; Siretanu, I. Correlation between electrostatic and hydration forces on silica and gibbsite surfaces: An atomic force microscopy study. *Langmuir* **2022**, *38* (3), 914-926.

(3) Klaassen, A.; Liu, F.; Van den Ende, D.; Mugele, F.; Siretanu, I. Impact of surface defects on the surface charge of gibbsite nanoparticles. *Nanoscale* **2017**, *9* (14), 4721-4729.

(4) Zhao, C.; Ebeling, D.; Siretanu, I.; van den Ende, D.; Mugele, F. Extracting local surface charges and charge regulation behavior from atomic force microscopy measurements at heterogeneous solid-electrolyte interfaces. *Nanoscale* **2015**, *7* (39), 16298-16311.

(5) Siretanu, I.; Ebeling, D.; Andersson, M. P.; Stipp, S. S.; Philipse, A.; Stuart, M. C.; Van Den Ende, D.; Mugele, F. Direct observation of ionic structure at solid-liquid interfaces: a deep look into the Stern Layer. *Scientific reports* **2014**, *4* (1), 4956.

(6) Israelachvili, J. N. *Intermolecular and surface forces*; Academic press, 2011.

(7) Kumar, S.; Cats, P.; Alotaibi, M. B.; Ayirala, S. C.; Yousef, A. A.; van Roij, R.; Siretanu, I.; Mugele, F. Absence of anomalous underscreening in highly concentrated aqueous electrolytes confined between smooth silica surfaces. *Journal of colloid and interface science* **2022**, *622*, 819-827.

(8) Behrens, S. H.; Borkovec, M. Electric double layer interaction of ionizable surfaces: Charge regulation for arbitrary potentials. *The Journal of chemical physics* **1999**, *111* (1), 382-385.

(9) Trefalt, G.; Behrens, S. H.; Borkovec, M. Charge regulation in the electrical double layer: ion adsorption and surface interactions. *Langmuir* **2016**, *32* (2), 380-400.

(10) Carnie, S. L.; Chan, D. Y. Interaction free energy between plates with charge regulation: a linearized model. *Journal of colloid and interface science* **1993**, *161* (1), 260-264.
